# Can power market reform reduce air pollution?——Evidence from prefecture-level cities in China

**DOI:** 10.1371/journal.pone.0282124

**Published:** 2023-04-13

**Authors:** Xing Li, Zimin Liu, Dan Yang, Yong Wei, Na Gong

**Affiliations:** 1 College of Economics and Management, Southwest University, Beibei District, Chongqing, China; 2 Institute for Rural Revitalization Strategy, Southwest University, Beibei District, Chongqing, China; 3 College of State Governance, Southwest University, Beibei District, Chongqing, China; 4 Chongqing Youth Vocational & Technical College, Beibei District, Chongqing, China; UCL: University College London, UNITED KINGDOM

## Abstract

The market-oriented reform of China’s power market has gradually transformed power prices from government pricing to market regulation, which not only promotes the production efficiency of industrial enterprises, but also inhibits the excessive consumption and waste of power by residential power users. This paper uses the data from 2006–2018 combined with the precious industrial power price data and macroeconomic data of 100 cities in China, takes the marketization reform of the power market in 2015 as a quasi-natural experiment, and uses the difference-in-differences model to empirically study the causal relationship between power market reform and air pollution for the first time. The study found that power market reform can reduce air pollution, and this conclusion is also supported by a number of robustness tests. Mechanism analysis shows that power market reform can reduce air pollution by improving power market efficiency, promoting technological progress, and reducing power consumption. Heterogeneity analysis shows that power market reform can suppress air pollution more significantly in eastern regions, regions with severe air pollution, and regions with larger populations. This paper not only provides new research perspectives and research ideas for air pollution prevention and control, but also provides empirical evidence for the positive externalities of power market reform.

## 1. Introduction

The report of the 19th National Congress of the Communist Party of China pointed out that it is necessary to "continue to implement air pollution prevention and control actions to win the battle to defend the blue sky", and to "build an environmental governance system led by the government, with enterprises as the main body, and social organizations and the public participating together." As a developing country, while China has achieved rapid economic growth, the air pollution situation is not optimistic. China’s environmental performance ranks 120th among 180 countries worldwide, and that air quality in less than 50 of all 500 large cities meets World Health Organization standards [1]. Especially in recent years, the severe haze weather that often occurs in some northern cities has seriously damaged the ecological environment, affecting industrial production and the health of residents. Ecological damage and air pollution can lead to three serious consequences, namely, reducing the comfort of human life, affecting the efficiency of industrial production, and affecting the health of residents. In order to reduce air pollution and improve the efficiency of economic operation and the happiness of residents’ life, China’s economy has shifted from high-speed growth to high-quality development in 2017, and has introduced a series of government policies to prevent and control air pollution.

In the process of controlling air pollution and achieving high-quality economic development, the relationship between the power industry and air pollution cannot be ignored. On the one hand, China has the world’s largest power system, with more installed capacity than all other countries combined. As China is a country with coal as its main energy consumption, thermal power (coal power) accounts for 70% of all power generation, making China’s power industry an industry with high air pollutant emissions [2]. On the other hand, the rapid development of China’s social economy has also led to a rapid increase in the demand for power from industrial and residential sectors, which has further led to air pollutants generated in the production, use and consumption of power.

The Chinese government’s pricing mechanism for power has further aggravated the emission of air pollutants from the power industry. The price of China’s power market is mainly set by the government, lacking the regulation of market mechanism. Since the 1970s, in order to further expand the use of power in households, the Chinese government has set a price that is far lower than the production cost, mainly considering the affordability of residents in the process of setting residential power prices [3]. With the rapid development of social economy and the great improvement of residents’ living standards, the power price of residents’ households still refers to historical level, administrative mechanism, social stability and other factors during the formulation process, thus maintaining a low price level. The Chinese government mainly subsidizes household power consumption by setting power prices far higher than production costs for industrial enterprises. For households, the low power price can not play a role in regulating power consumption, which leads to excessive power consumption and waste of power, resulting in a large number of air pollutants; For the industrial sector, higher power prices crowd out technological innovation and inhibit production efficiency, which will also lead to large emissions of air pollutants in the production process [4].

Theoretically, the market-oriented reform of China’s power market can reduce air pollution emissions. The main means of China’s power market reform are to reduce industrial power prices and increase residential power prices. For industrial enterprises, the reduction of industrial power price reduces the crowding out of R&D investment, enables industrial enterprises to achieve production technology innovation [5], thus reducing the emission of air pollutants; For households, the increase of power price can restrain the rich from using excessive power and wasting power, thus reducing the emission of air pollutants [6]; For the whole power market, the marketization and rationalization of power price can make the resource allocation reasonable, improve the operation efficiency of the power market, and thus reduce the emission of air pollutants [7].

In order to study the causal relationship between power market reform and air pollution, this paper merges precious industrial power prices and macroeconomic data from 100 prefecture-level cities in China from 2006 to 2018, and takes the Document No. 9 implemented in 2015 as a quasi-natural experiment, using the difference-in-differences (DID) model to study the effect and mechanism of power market reform on air pollution characterized by *PM*2.5 concentration. The study found that power market reform can reduce air pollution, and this conclusion is also supported by multiple robustness tests. The results of the mechanism analysis show that the power market reform mainly suppresses air pollution by improving the operating efficiency of the power market, promoting the level of technological progress and reducing power consumption. Heterogeneity analysis shows that power market reform is more effective in reducing air pollution in eastern regions, regions with severe air pollution, and regions with larger populations. The research in this paper not only provides new research perspectives and research ideas for China’s air pollution prevention and control, but also provides empirical evidence for the positive externalities of the market-oriented reform of China’s power market.

The marginal contribution of this paper mainly exists in the following three aspects: First, this paper is among the first to study the causal relationship between China’s power market reform and air pollution. In the process of studying topics related to air pollution, existing literatures usually focus on the impact of government regulations and environmental policies on air pollution [8, 9]. This paper focuses for the first time on the impact of the market-oriented reform of the power market on air pollution.

Second, the precious city level power market data also enables this paper to more accurately study the impact of power market reform on air pollution. Due to the lack of city-level power price data, the literature usually makes a rough policy evaluation of power market reform by setting dummy variables, and there is no way to study the impact of price changes in the power market on air pollution in detail. This paper accurately identifies the causal relationship between power market reform and air pollution by using precious city level power price data.

Third, we empirically studied the mechanisms of power market reform on air pollution. The existing literature more theoretically describes the mechanism of power market reform on air pollution [10], while this paper conducts an empirical test on the mechanism of power market reform affecting air pollution through the collection of city level data.

The arrangement of the research content of this paper is as follows: Chapter 2 is the literature review on power market reform and air pollution; Chapter 3 is the empirical design; Chapter 4 is the empirical results and discussion; Chapter 5 is the analysis of the mechanism of power market reform affecting air pollution; Chapter 6 is the heterogeneity analysis of the impact of power market reform on air pollution; Chapter 7 is the conclusion and policy recommendations.

## 2. Policy background and literature review

### 2.1. Power market reform in China

The power market includes industrial enterprises and households. During the promotion period of power, the Chinese government can indeed promote power services by setting an industrial power price higher than the cost to subsidize the price of power for residents (thus making the price of power for residents far lower than the cost). However, with the rapid development of China’s economy and society, and the gradual saturation of power services, the drawbacks of government pricing are gradually exposed. According to market experience such as power cost, industrial power price should be lower than residential power price. However, the pricing of the Chinese government makes the industrial power price higher than the residential power price, resulting in the crowding out of R&D investment and technological innovation of industrial enterprises, as well as excessive power consumption and waste of power by households. The reduction of production efficiency of industrial enterprises and the unreasonable use of power by households have resulted in a large amount of air pollutants from China’s power market, where coal is the main energy consumption.

In order to improve the operation efficiency of the power market and reduce the emission of air pollutants, the Chinese government carried out the power market reform for the first time in 2002. Although this reform did not achieve the goals of improving the operation efficiency of the power market and reducing air pollution emissions, it also accumulated a lot of experience. Since 2015, the Chinese government has reformed the price mechanism of the power market again, making the Chinese power market begin to change from government pricing to market mechanism. In view of the misplacement of industrial power price and residential power price in China’s power market, the power market reform in 2015 included a series of market-oriented reform measures, such as gradually reducing the power price of industrial enterprises, and gradually increasing the power price of residents by Increasing Block Pricing (IBP), so that the price of power used by industrial enterprises and the residents will gradually return to their cost, and the price mechanism will effectively regulate the supply and demand of power. In addition to the direct adjustment of industrial power prices and residential power prices, the Chinese government has also coordinated the implementation of power market, carbon market, Green Certificate and other environmental protection policies, so as to gradually integrate the cost of environmental governance into power prices.

### 2.2. Literature review and theoretical mechanisms

A large number of air pollutants are discharged in the power market, which is a problem faced by most countries in the world in the process of environmental pollution control [11]. As the most important secondary energy, the huge demand for power and the characteristics of coal consumption make the power market emit a lot of air pollutants in the process of operation [12, 13]. China is a country with coal as its main energy consumption. 70% of the power generation in the power market is generated by the consumption of coal. In addition, the production equipment with low efficiency makes China’s power market emit a lot of air pollutants in the process of producing and consuming power [14]. With the rapid development of China’s social economy, both industrial enterprises and households are rapidly increasing their demand for power, which has aggravated the emission of air pollutants in China’s power market. More importantly, the power prices of industrial enterprises and households in China’s power market are set by the government according to the residents’ affordability, historical level, administrative mechanism, social stability and other aspects, not determined by the market mechanism, which cannot fully reflect the level of power supply and demand of industrial enterprises and households [15]. The abnormally high price of power used by industrial enterprises and the low price of power used by households distort the price of power in China’s power market, resulting in deadweight losses such as resource mismatch, reduced operating efficiency, and backward technological progress, which further aggravates the emission of air pollutants.

China’s power marketization reform is committed to reducing the degree of price dislocation and price distortion in the power market by reducing the price of power used by industrial enterprises and increasing the price of power used by residents, so that the price of power used by industry and residents can reflect their own power costs and the relationship between supply and demand [16] Through the market-oriented reform, the price signal in China’s power market can correctly reflect the level of power supply and demand of industrial enterprises and residents, so as to play the role of regulating power consumption [17]. In addition, price dislocation and price distortion are gradually corrected, which can play a correct role in guiding the flow of production resources, so as to make the best use of everything, and ultimately improve the operation efficiency of China’s power market and reduce air pollutant emissions [18]. More importantly, China’s power market will be combined with the carbon market policy and renewable energy power generation policy in the process of reform, so as to more curb the emission of air pollutants [19]. Novan [20] quantified the heterogeneity in the marginal impact of renewable energy generation on air pollution using hourly changes produced by wind turbines. He found that increased renewable power generation capacity can provide different marginal external benefits, resulting in lower air pollution. Fujii et al. [21] took China’s industrial sector from 1998 to 2009 as an example to study the relationship between the management of the power industry and air pollution. They found that although *PM*2.5 emissions increased with production scale, *PM*2.5 emissions from China’s power sector dropped by 65% during the sample period due to improvements in energy efficiency and end-of-line treatment technologies. Kaygusuz [22] analyzed the air pollution problems in the energy production and consumption process in the power industry in Turkey. The study found that the implementation of the renewable energy policy is the most effective policy for achieving clean and sustainable development in Turkey. Based on the above discussion, we formulated our hypotheses:

Hypothesis 1. Power market reform can restrain the emission of air pollutants.
Hypothesis 2. Power market reform can restrain air pollution emissions by improving the operation efficiency of the power market.

For industrial enterprises in the power market, the high price of industrial power set by the government makes industrial enterprises need to pay more power costs, thus reducing R&D investment and investment in technological innovation activities when the profitability of industrial enterprises remains unchanged [23]. In other words, the excessive industrial power price set by the government has squeezed out the technological innovation activities of industrial enterprises [24]. With the reform of power market, the price of industrial power is gradually reduced and approaching the cost level [25]. The reform of power marketization has reduced the cost of power for industrial enterprises, enabling more R&D investment and technological innovation. The innovation of production technology can improve production efficiency, thus reducing the emission of air pollutants in the industrial production process. Yue et al. [26] studied the benefits of improved industrial efficiency and clean energy power systems in China’s coal-intensive power supply system. They developed a comprehensive model framework that quantified the relationship between power savings, coal-fired power systems, and air pollution, finding that improvements in industrial efficiency can significantly reduce reliance on coal-fired power generation and significantly improve air quality. Yue et al. [27] quantified the impact of industrial power savings on the evolution of the number of coal-fired power plants and air pollutants in China from 2016 to 2040 by developing an integrated model framework, finding that improvements in industrial efficiency can shut down the most polluting units, and cause the power grid to reduce air pollutants such as *PM*2.5, nitrogen oxides, and sulfur dioxide by more than 100,000 tons per year. Jiang et al. [28] studied the impact of China’s power industry on carbon dioxide and air pollutant emissions. They found that technological advances and structural transformation and upgrading in the power industry can achieve synergistic reductions in carbon dioxide and air pollutant emissions, but operating air pollution control devices can lead to increased carbon emissions. Based on the above discussion, we formulated our Hypothesis 3:

Hypothesis 3. Power market reform can curb air pollution emissions by promoting technological progress.

On the one hand, the improvement of the operation efficiency of the power market and the technological progress in the production process of the industrial sector can achieve less air pollutants under the same economic output; on the other hand, for households in the power market, the residential power price set by the government is far lower than the power cost. Especially with the rapid development of China’s economy and society, the low price of residential power can not form a reasonable regulatory effect on the power consumption behavior of residential households, so there are a lot of excessive power consumption and waste of power in residential households [29]. The consumption of a large amount of power energy makes the entire power industry chain emit huge air pollutants. With the reform of the power market, the price of power for households has gradually risen to the cost level, which effectively curbs the behavior of high income households that use excessive power and waste power [30]. The reform of the power market is also combined with the price mechanisms such as the IBP of residents and the time of use price of peak and valley, which effectively promotes the staggered and rational use of power by residents. Based on the above discussion, we formulated our Hypothesis 4:

Hypothesis 4. Power market reform can restrain air pollution emissions by reducing power consumption.

## 3. Empirical strategy

### 3.1. Econometric model construction

In order to eliminate the endogeneity problem in the empirical model and more accurately evaluate the causal relationship between the power market reform and *PM*2.5, this paper draws on the relevant research of [31] and other related studies, and sets up the following DID model:

$$PM2.5_{it}=\beta_{0}+\beta_{1}Year_{2015}\times Power_{i}+\beta_{2}Control_{it}+\beta_{3}\delta_{i}+\beta_{4}\gamma_{t}+\varepsilon_{it}$$ (1)

where the subscript *i* is the city, and *t* is the year. *PM*2.5_*it*_ represents the level of air pollutants in city *i* in year *t*, and is characterized by the concentration of fine particulate matter in the city. *Year*_2015_ is the difference in the time dimension, which is used to distinguish whether China’s power marketization reform has been implemented. Since the reform of China’s power market took place in 2015, we set *Year*_2015_ before 2015 equal to 0, indicating that the policy did not take place. We set *Year*_2015_ after 2015 equal to 1, indicating that the power market reform has been implemented. *Power*_*i*_ represents the difference in the city dimension. Since the power marketization reform policy has been implemented nationwide since 2015, there is no treatment group and control group in the traditional sense.

The power market reform started in 2015 mainly focused on reducing industrial power prices, while residential power prices remained almost unchanged (the reform of residential IBP was completed in 2012). Therefore, in order to better study the impact of power marketization reform on air pollution, this paper draws on the ideas of Qian [32], and selects the industrial power price directly affected by power marketization reform as an indicator to construct a treatment group and a control group. Specifically, because the main way of power marketization reform is to reduce the industrial power price, the higher the industrial power price, the greater the reduction of the industrial power price, and the greater the influence of the power marketization reform policy. Therefore, this paper sorts all sample cities according to the industrial power price from high to low, and sets the top 50% of the cities with higher industrial power prices as the treatment group, and the last 50% of the cities with lower industrial power prices as the control group. *Power*_*i*_ represents the industrial power price for each city. *Control*_*it*_ represents a group of control variables at the city level, including variables such as economic growth, population density, and industrial structure. *δ*_*i*_ is the city fixed effect, *γ*_*t*_ is the year fixed effect, and *ɛ*_*it*_ is the random disturbance term. *β*_1_ is the core coefficient of concern in this paper, which represents the impact of the implementation of power market reform on air pollution.

### 3.2. Main indicators

#### 3.2.1. Air pollution (*PM*2.5)

This paper uses the *PM*2.5 concentration data in the air to characterize the air pollution level. The higher the *PM*2.5 concentration, the more serious the air pollution level. The *PM*2.5 concentration data used in this paper comes from the processing of satellite remote sensing data by the Atmospheric Composition Analysis Group (ACAG) [33]. This paper extracts the layer information in NetCDF format, and matches it with the macroeconomic data of each prefecture-level city through regional statistics. Finally, 1300 observations of *PM*2.5 concentrations in all 100 cities were obtained.

#### 3.2.2. Market-oriented reform of power market (*Year*_2015_*Power_*i*_)

As mentioned above, since the power market reform took place in 2015, this paper assigns all *Year*_2015_ after 2015 as 1, indicating that the power market reform occurred in these years. We also assign a value of 0 to all Year2015 before 2015, indicating that no power market reform occurred in these years. As the power market reform is implemented uniformly throughout the country, it is not easy to distinguish between the treatment group and the control group at the urban level. Qian [32] proposed an improved DID model in response to this situation, and believed that when the traditional DID model could not effectively divide the region into treatment group and control group, we could find variables closely related to the policy and divide the region into treatment group and control group according to the degree of correlation. Considering that the core means of China’s power market reform is to reduce the price of power used by 10% of industrial enterprises, the higher the industrial price is, the higher the regional price will fall, which can better reflect the effect of the power market reform policy. According to Qian [32], the first 50% of cities with higher industrial power prices are set as treatment groups (Poweri = 1), while the last 50% of cities with lower industrial power prices are set as control groups (Poweri = 0). Similar practices can also refer to VIG [34] and Campello and Larrain [35].

#### 3.2.3. Control variables

In order to exclude the impact of other variables on *PM*2.5 concentration, so as to obtain a pure causal relationship between power market reform and *PM*2.5 concentration, this paper controls three types of variables, namely macroeconomic variables, industrial variables and weather variables.

1. macroeconomic variables. The relationship between macroeconomic variables and *PM*2.5 concentration has been confirmed by most literatures [36]. For example, the more population in a region, the more *PM*2.5 it emits in the production and living process; In addition, the larger the area is, the more people and factories there are, so the more *PM*2.5 is emitted; The per capita GDP is also closely related to the concentration of *PM*2.5. The higher the per capita GDP is, the more vigorous the industrial production activities are, and the more *PM*2.5 is emitted. In order to control the impact of these macroeconomic variables on the estimated results, we have controlled variables such as population with registered permanent residence at the end of the year (*RP*, 10,000 people), administrative area (*AA*, square kilometer) and per capita GDP (*GDP*, yuan/person).
2. industrial variables. Industrial variables are directly related to *PM*2.5 concentration [37]. The higher proportion of secondary industry employees, the higher proportion of secondary industry output value, the more industrial enterprises, and the higher total industrial output value in a region all indicate that the manufacturing industry in this region is more developed. Under China’s extensive development model, developed industries also mean more *PM*2.5 emissions. In addition, the more industrial water consumption, industrial gas consumption and industrial petroleum gas consumption, the more active industrial production activities in the region will also generate more *PM*2.5. In order to control the impact of industrial variables on the estimated results, we have controlled variables such as the employee proportion in the secondary industry (*ESI*, %), the proportion of the output value of the secondary industry (*OSI*, %), the number of industrial enterprises (*IE*), the total industrial output value (*IO*, 100 million yuan), the consumption of industrial water (*IW*, 10,000 tons), the consumption of industrial gas (*IG*, 100 million cubic meters) and the consumption of industrial petroleum gas (*IP*, 10,000 tons). More importantly, this paper also controls the residential power price (*RPP*, yuan) considering that the it may have an impact on the industrial power price and *PM*2.5 [38]. The above data is collected from *the China City Statistical Yearbook*.
3. weather variables. Weather variables such as average temperature and average humidity have been proved to affect *PM*2.5 concentration [39]. Is paper controls the weather factors that could significantly affect *PM*_2.5_ such as the average temperature (*AT*, 0.1°C) and the average humidity (*AH*, %) [40]. The weather data is collected from *the China Meteorological Yearbook*.

**Table 1** shows the descriptive statistics of all variables from 2006 to 2018. During the sample period, the average of annual *PM*2.5 reaches 39.7464μg/m^3^ and the standard deviation is 20.6788 which indicates that the *PM*2.5 in different cities vary greatly in different years. This provides statistical evidence and logical support for this study to use the DID model. Furthermore, the mean value of *Year*_2015_*Power_*i*_ is 0.1476, the minimum value is 0, and the maximum value is 0.9267, which also provides a sufficient sample for this paper to compare and analyze the differences between the treatment group and the control group.

**Table 1:** Descriptive statistics.

Variable	Obs	Mean	S.D.	Min	Max
*PM*2.5	851	46.315	17.950	3.5455	99.515
*Year*_2015_*Power_*i*_	851	0.1476	0.3102	0	0.9267
*RP*	851	565.48	431.13	43.350	3392
*AA*	851	14453	11926	845	82829
*GDP*	851	43729	35429	1.7486	467749
*ESI*	851	47.372	13.168	9.8500	84.400
*OSI*	851	49.012	9.9746	18.570	85.640
*IE*	851	2102.3	2540.2	19	18792
*IO*	851	4986.9	6052.0	20.176	32445
*IW*	851	21510	31484	316	256236
*IG*	851	5.9923	18.681	0	195.99
*IP*	851	3.5371	7.9036	0	75.300
*RPP*	851	0.5283	0.05530	0.4150	0.7600
*AT*	851	147.86	49.302	42	254
*AH*	851	65.530	9.9185	38	84

## 4. Analysis of empirical results

### 4.1. Parallel trend test

In order to test the applicability of the DID model in this paper, and to show that the estimation results are convincing, a parallel trend test is carried out in this paper. Specifically, in order for the baseline regression results to accurately reflect the impact of the power market reform on *PM*2.5 concentration, this paper needs to ensure that before the implementation of the power market reform in 2015, the change trend of *PM*2.5 concentration in the treatment group and the control group is keep it parallel. Only in this way can we be sure that the difference in *PM*2.5 concentration change trend between the treatment group and the control group after 2015 was caused by power market reform. To this end, this paper conducts a parallel trend test through the following model.

$$PM2.5_{it}=\phi_{0}+\sum \tau \phi_{\tau }Year_{\tau }*Power_{i}+\phi_{1}Control_{it}+\phi_{2}\delta_{i}+\phi_{3}\gamma_{t}+\varepsilon_{it}$$ (2)

where the subscript *i* is the city, and *t* is the year. *PM*2.5_*it*_ represents the air pollution level of city *i* in year *t*. *τ*∈{2013-, 2014, 2015, 2016, 2017+}, *Year*_τ_*Power_*i*_ represents the set of dummy variables for power market reform in 2013 and before, 2014, 2015, 2016, 2017 and later. *Control*_*it*_, *δ*_*i*_, *γ*_*t*_, and *ɛ*_*it*_ denote control variables, city fixed effects, year fixed effects, and random error terms, respectively. *ɸ*_*τ*_ is the coefficient concerned in this paper, which characterizes the time trend of the difference in *PM*2.5 concentration between the treatment group and the control group before and after the implementation of the power market reform.

**Fig 1:**
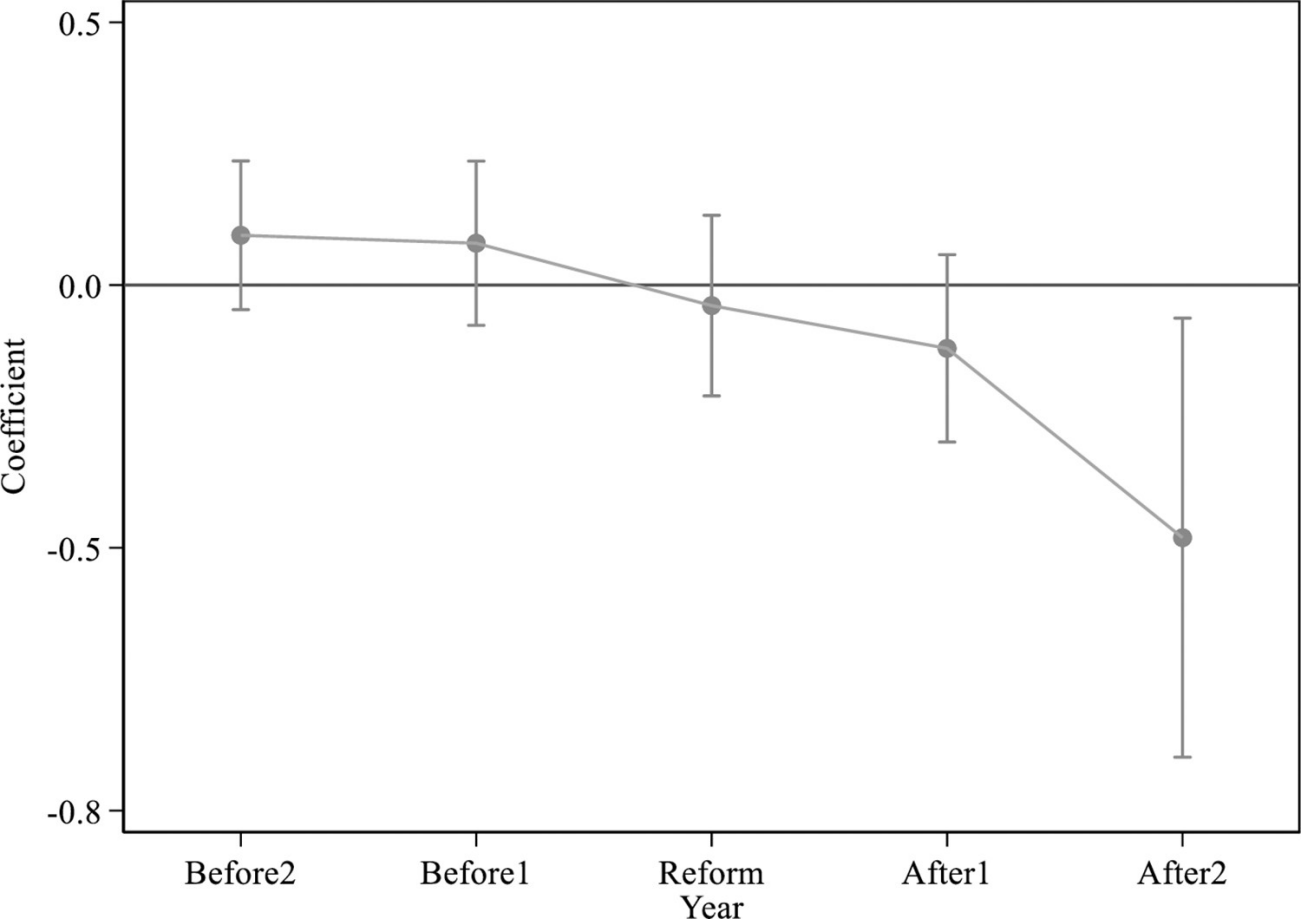
Parallel trend test.

Fig 1 reports the results of a parallel trend test using model (2). It can be seen that the estimated coefficients are not significantly different from 0 in all years before the implementation of the power market reform policy in 2015, which shows that before the implementation of the power market reform, the change trend of *PM*2.5 concentration in the treatment group and the control group is parallel and there is no difference. Therefore, the research in this paper has passed the parallel trend test, which proves that the estimation results obtained by using the DID model in this paper are accurate and unbiased.

### 4.2. Analysis of baseline regression results

This paper uses model (1) to estimate the impact of power market reform on air pollution. The estimated results are shown in Table 2. Column (1) controls only city fixed effects and year fixed effects, and column (2) controls macroeconomic variables at the city level, including population, area, and GDP. Column (3) further controls for industry-related variables, and column (4) further controls for weather variables based on column (3). From the results reported in Table 2, it can be seen that with the increase of control variables, the inhibitory effect of power market reform on air pollution is gradually strengthened. After controlling for all control variables (column (4)), the power market reform was able to reduce the *PM*2.5 concentration in the air by 0.54%.

**Table 2:** Baseline regression results.

	*PM*2.5			
	(1)	(2)	(3)	(4)
*Year*_2015_*Power_*i*_	-0.2305***	-0.3817***	-0.4867***	-0.5401***
	(0.0464)	(0.0526)	(0.1472)	(0.1572)
*RP*		0.3757***	-0.2752*	-0.2849**
		(0.0709)	(0.1411)	(0.1340)
*AA*		-0.2208***	0.0850	0.0987
		(0.0737)	(0.0553)	(0.0606)
*GDP*		-0.0680	-0.0062	-0.0055
		(0.0424)	(0.0064)	(0.0060)
*ESI*			0.1162	0.1083
			(0.0763)	(0.0745)
*OSI*			0.0753	0.0762
			(0.1400)	(0.1329)
*IE*			0.0169	0.0111
			(0.0427)	(0.0441)
*IO*			-0.0841	-0.0757
			(0.0512)	(0.0520)
*IW*			-0.0101	-0.0134
			(0.0181)	(0.0178)
*IG*			-0.0123	-0.0132
			(0.0089)	(0.0087)
*IP*			0.0025	0.0037
			(0.0046)	(0.0044)
*RPP*			0.0530	0.0550
			(0.2093)	(0.2024)
*AT*				-0.0002
				(0.0009)
*AH*				-0.0048***
				(0.0014)
Constant	2.7296***	4.2258***	3.0178***	3.4235***
	(0.0147)	(0.8251)	(0.6843)	(0.7129)
Adjusted R-squared	0.9342	0.3771	0.9229	0.9244
Observations	851	851	851	851
Cities	92	92	92	92

Note: The cluster robust standard errors at the city level are in brackets

*, **, *** indicate significance at the 10%, 5%, and 1% levels, respectively. The following tables are the same.

The possible explanation is that, on the one hand, China’s power market reform can transform the power pricing mechanism from government pricing to market regulation, so that the power price signals of industrial and residential sectors can increasingly reflect the power cost. With the growing role of the market mechanism, the mismatch of resources has been reduced, and under the regulation of the supply and demand relationship, the operating efficiency of China’s power market has been continuously improved, which can reduce air pollutant emissions. On the other hand, the decline of industrial power price enables the industrial sector to invest more funds in technology research and development. With the continuous progress of production technology, the air pollution properties emitted by the production or use of the same power gradually reduce. Finally, the improvement of the operation efficiency of China’s power market and the progress of production technology also reduce the power consumption in the production process, which also means that thermal power generation and the air pollutants generated by thermal power generation are reduced. These results provide supportive evidence for Hypothesis 1.

### 4.3. Robustness check

#### 4.3.1. Placebo test

In order to exclude the interference of other policies and their lag effects on the baseline regression results of this paper and obtain more convincing conclusions, this paper conducts a placebo test in the time dimension. Specifically, although the DID model is used in this paper, the endogeneity problem of the model is largely excluded. However, this paper obtains the effect of power market reform on *PM*2.5, which may be mixed with the effects of other policies and their lag effects. In order to make the estimation of *PM*2.5 concentration by the power market reform more robust, this paper advances the time of power market reform through the construction of counterfactual scenarios, assuming that it happened in 2014, 2013 and 2012 respectively. If the power market reform in the counterfactual scenario can still significantly reduce *PM*2.5 concentration, it means that the decrease in *PM*2.5 concentration in the baseline regression results may be caused by other policies or their lag effects. If it is not significant, it means that the baseline regression results are robust. From the results reported in Table 3, after advancing the policy time, the inhibitory effect of power market reform on *PM*2.5 concentration is no longer significant. It shows that the baseline regression results are convincing.

**Table 3:** Placebo test.

	One year	Two years	Three years
	(1)	(2)	(3)
*Year*_2015_*Power_*i*_	-0.5092	-0.4149	0.4494
	(0.5316)	(0.3316)	(0.3324)
Control variables	YES	YES	YES
City fixed effect	YES	YES	YES
Year fixed effect	YES	YES	YES
Constant	0.0193	0.1093*	0.0994
	(0.0401)	(0.0602)	(0.0634)
Adjusted R-squared	0.9309	0.9309	0.9309
Observations	851	851	851
Cities	92	92	92

#### 4.3.2. Replace the independent variable

In model (1), this paper divides the top 50% cities with higher industrial power prices into the treatment group according to the level of industrial power prices, and the rest as the control group. In order to classify the treatment group and the control group in a more detailed manner, in the column (1) of Table 4, this paper takes the top 30% of the highest industrial power price as the treatment group, and assigns it a value of 1, and the rest of the cities are assigned a value of 0 for regression; in column (2), the top 30% with the highest industrial power price is used as the treatment group, and the 30% with the lowest industrial power price is used as the control group for regression. From the results reported in columns (1) and (2) of Table 4, it can be seen that the conclusion that power market reform can inhibit *PM*2.5 concentration is robust. It should be emphasized that although we have studied the causal relationship between the power market reform and *PM*2.5 concentration through the improved DID model based on the methods of Qian [32], the use of dummy variables will erase the effective information related to power prices in the power market reform to a certain extent, so as to obtain a relatively rough estimation result. In order to more rigorously and reasonably explore the causal relationship between the power market reform and *PM*2.5 concentration, this paper uses the time dummy variable (Year2015) to multiply with the power price and the degree of price decline, respectively, and displays the results in the (3) and (4) columns of Table 4. It can be found that after using the real price information for regression, the power market reform can still suppress the *PM*2.5 concentration at the significance level of 1%, which shows that the results of the benchmark regression are convincing.

**Table 4:** Robustness test based on the setting of treatment groups.

	Top 30%	Drop the middle 30%	Industrial power price	Degree of price reduction
	(1)	(2)	(3)	(4)
*Year*_2015_*Power_*i*_	-0.8427***	-0.5201***	-0.2315***	-0.0431***
	(0.2228)	(0.0566)	(0.0067)	(0.0022)
Control variables	YES	YES	YES	YES
City fixed effect	YES	YES	YES	YES
Year fixed effect	YES	YES	YES	YES
Constant	1.2354***	1.6748***	0.9281***	0.0469***
	(0.2178)	(0.2572)	(0.0034)	(0.0024)
Adjusted R-squared	0.9544	0.9551	0.9662	0.9599
Observations	851	511	851	851
Cities	92	56	92	92

#### 4.3.3. Replace the dependent variable

This paper also conducts robustness test by replacing the dependent variable. First of all, the data used in this paper comes from the processing of satellite remote sensing data by the Atmospheric Composition Analysis Group (ACAG), and the *PM*2.5 data is the average within one year. In order to test the robustness of the results, this paper replaces the data in column (1) of Table 5 with the *PM*2.5 concentration data obtained after three-year moving average processing of satellite data by Columbia University. Since the *PM*2.5 concentration data from Columbia University was updated to 2016, this paper uses interpolation to fill in the data for 2017 and 2018. Secondly, this paper also replaces the original *PM*2.5 concentration data with per capita *PM*2.5 concentration and *PM*2.5 emission intensity in columns (2) and (3) respectively, in order to exclude the influence of the population and economic development in the city on the *PM*2.5 concentration. Finally, because China is a country dominated by coal energy, coal will emit a large amount of greenhouse gases such as carbon dioxide and air pollutants such as *PM*2.5 during the combustion process. *PM*2.5 and carbon dioxide have the same root homology and the synchronization of pollution. This paper uses the CO_2_ emissions data to substitute *PM*2.5 in column (4). In general, no matter what way to replace the *PM*2.5 concentration data, the inhibitory effect of power market reform on *PM*2.5 is still significant.

**Table 5:** Replacement dependent variables.

	*PM*2.5 (Columbia)	*PM*2.5 per capita	*PM*2.5 intensity	CO_2_
	(1)	(2)	(3)	(3)
*Year*_2015_*Power_*i*_	-0.8484***	-0.6748**	-0.7928***	-0.6521**
	(0.2477)	(0.2831)	(0.2395)	(0.2985)
Control variables	YES	YES	YES	YES
City fixed effect	YES	YES	YES	YES
Year fixed effect	YES	YES	YES	YES
Constant	-0.0615***	1.5709***	1.6356***	2.1179***
	(0.0017)	(0.2651)	(0.2756)	(0.3438)
Adjusted R-squared	0.9199	0.9328	0.9602	0.9018
Observations	851	851	851	851
Cities	92	92	92	92

#### 4.3.4. Replace the model

In the process of model setting, this paper controls the variables closely related to *PM*2.5 concentration as comprehensively as possible from the aspects of macro economy, industry and weather. We also controlled for the city fixed effect and year fixed effect, and clustered the standard errors to the city level, in order to more accurately assess the impact of power market reform on *PM*2.5 concentrations. However, the model may still have some degree of missing variables. In order to obtain the impact of power market reform on *PM*2.5 concentration more accurately, this paper adds the multiplication term of city fixed effect and year fixed effect in column (1) of Table 6 to control the characteristics of cities in different years. This paper also controls the multiplication term of the province fixed effect and the year fixed effect in column (2) to control the influence of the characteristics of each province on the *PM*2.5 concentration in different years. Finally, this paper uses PSM-DID in column (3) to regress the effect of power market reform on *PM*2.5 concentrations. From the results reported in Table 6, power market reforms are still able to reduce *PM*2.5 concentrations after replacing the estimated model.

**Table 6:** Alternative models.

	City×Year FE	Province×Year FE	PSM-DID
	(1)	(2)	(3)
*Year*_2015_*Power_*i*_	-0.5920***	-0.5183***	-0.7832***
	(0.0265)	(0.0693)	(0.0367)
Control variables	YES	YES	YES
City fixed effect	YES	YES	YES
Year fixed effect	YES	YES	YES
Constant	1.4235***	1.3167***	2.4788***
	(0.3220)	(0.3281)	(0.3961)
Adjusted R-squared	0.9271	0.9368	0.9226
Observations	851	851	782
Cities	92	92	87

#### 4.3.5. Eliminate interference from environmental policies

(1) Air pollution prevention and control policies. Since 2013, the Chinese government has introduced a series of air pollution prevention and control measures in order to reduce the concentration of air pollutants and improve air quality. For example, the Chinese government deployed ten air pollution prevention and control measures in 2013 to save energy and reduce air pollutant emissions. In 2017, the Ministry of Environmental Protection designated Beijing and 28 nearby cities as key areas for air pollution prevention and control in autumn and winter. These air pollution control policies work in areas with the most air pollution and aim to improve local air quality. Since these air pollution control policies can directly reduce the concentration of pollutants in the air, the inhibitory effect of the power market reform on air pollution in this paper will be overestimated. In order to exclude the impact of air pollution control policies on the estimation results of this paper, this paper excludes the 2+26 cities with the most serious air pollution and the strictest implementation of air pollution control policies in column (1) of Table 7. From the results reported in column (1), after excluding the impact of air pollution prevention and control policies, power market reform can still significantly reduce *PM*2.5 concentrations and improve air quality.

**Table 7:** Elimination of interference from environmental policies.

	Severe air pollution	Carbon market pilot	Low carbon pilot city
	(1)	(2)	(3)
*Year*_2015_*Power_*i*_	-0.8301***	-0.7514***	-0.7871***
	(0.2452)	(0.2109)	(0.2143)
Control variables	YES	YES	YES
City fixed effect	YES	YES	YES
Year fixed effect	YES	YES	YES
Constant	3.5113***	2.9777***	4.8890***
	(0.8255)	(0.7738)	(1.1877)
Adjusted R-squared	0.9205	0.9235	0.9274
Observations	755	741	518
Cities	82	80	54

(2) Low-carbon policies. In order to successfully achieve carbon peaking in 2030 and carbon neutrality in 2060, the Chinese government has gradually established seven carbon market pilot areas since 2013, and three batches of low-carbon pilot cities since 2010. The carbon market pilot mainly regulates the carbon emissions in the production process of industrial enterprises in the region, while the low-carbon pilot cities put forward more comprehensive carbon emission reduction requirements in various fields such as low-carbon economy, low-carbon production and low-carbon consumption. The implementation of carbon market pilot areas and low-carbon pilot cities has effectively reduced carbon emissions, which will lead to overestimation of the estimated results of the power market reform to suppress *PM*2.5 concentration. This paper deletes the carbon market pilot areas and low-carbon pilot cities in columns (2) and (3) of Table 7, respectively, in order to exclude the bias caused by the government’s carbon emission reduction policy on the results. It can be seen that after the carbon market pilot policy and the low-carbon pilot city policy are deleted, the power market reform can still significantly curb *PM*2.5.

## 5. Mechanism analysis

Combined with the previous research and existing literature, this paper believes that the reasons for the decline of regional *PM*2.5 caused by the power market reform mainly include the following aspects. Firstly, the market-oriented reform of China’s power market aims to make the price of industrial and residential power reflect the corresponding cost, reduce the cross-subsidy between the industrial power price and the residential power price in the power industry, and improve the operation efficiency of the power market. Therefore, the improvement of operation efficiency can reduce the use of energy such as coal in the power industry and the emission of *PM*2.5.

Secondly, the market-oriented reform of the power market makes the market mechanism in the power industry play an increasingly important role. In order to reduce production costs and obtain greater production profits, some enterprises will improve the level of production technology by updating production equipment and increasing investment in research and development [41, 42]. The improvement of production technology level can reduce the consumption of fossil energy, so as to achieve the effect of reducing *PM*2.5 emissions.

Finally, the residential power price in China is mainly determined by the government department based on factors such as historical levels, residents’ affordability, government administrative mechanisms, and social stability, rather than the cost of residential power consumption. Therefore, the power price in the residential sector is much lower than the cost. The extremely low price of residential power has caused some residents to overuse power, which increases the consumption of energy exchange and *PM*2.5 emissions from the perspective of the entire power industry. The power market reform aimed at raising the power price of residents can effectively curb residents’ excessive consumption of power and waste power, and ultimately reduce the concentration of *PM*2.5. Therefore, this paper draws on the research ideas of Li and Wang [39], adopts the classic mechanism analysis method in economics, and conducts mechanism test by setting the following model.

$$Mec_{it}=\theta_{0}+\theta_{1}Year_{2015}\times Power_{i}+\theta_{2}Control_{it}+\theta_{3}\delta_{i}+\theta_{4}\gamma_{t}+\varepsilon_{it}$$ (3)

where *Mec*_*it*_ represents the mechanism variables studied in this paper, including power market efficiency, technological progress, and power consumption. The meanings of other variables are the same as in model (1).

### 5.1. Operational efficiency of power market

This paper firstly examines the mechanism of power market efficiency in the process of power market reforms suppressing *PM*2.5 concentrations. Due to the serious lack of power-related cost information (such as power generation cost, power consumption cost, etc.) in China’s power industry, it is impossible to calculate the real operating efficiency of China’s power market through power cost data. Therefore, in order to study the mechanism of power market efficiency, this paper draws on the ideas of Hahn and Metcalfe [43], uses the precious industrial and residential power consumption and power price data to calculate the minimum value of the annual power market operating efficiency in each city. And use this to characterize the annual power market operation efficiency of each city.

In this paper, the calculated power market operation efficiency data is put into model (3) for regression, and the results are shown in Table 8. Among them, column (1) only studies the impact of power market reform on the operation efficiency of the power market, column (2) adds macroeconomic variables, and column (3) further increases the industry on the basis of column (2). variable. Column (4) controls all control variables. It can be seen that no matter whether the control variable is increased or not, the power market reform can significantly improve the operation efficiency of the power market, thereby achieving the effect of reducing *PM*2.5 concentration, which remains consistent with the Hypothesis 2.

**Table 8:** Impact of power market reform on efficiency.

	Efficiency			
	(1)	(2)	(3)	(4)
*Year*_2015_*Power_*i*_	0.1773**	0.2242***	0.2138***	0.2104***
	(0.0854)	(0.0682)	(0.0682)	(0.0623)
Control variables	NO	YES	YES	YES
City fixed effect	YES	YES	YES	YES
Year fixed effect	YES	YES	YES	YES
Constant	0.1149	0.1185*	0.0621	0.0994
	(0.0700)	(0.0685)	(0.0869)	(0.0634)
Adjusted R-squared	0.8987	0.8843	0.8692	0.9158
Observations	851	851	851	851
Cities	92	92	92	92

### 5.2. Technological progress

Since the market-oriented reform of the power market enables the market mechanism and price mechanism to fully play a role, some industrial enterprises will create more output with less input by updating production equipment and increasing R&D investment, thereby reducing production costs and increasing production profits. In order to investigate the mechanism and role of technological progress in the process of power market reform affecting *PM*2.5 concentration, this paper uses five variables that are closely related to technological progress and innovation output, such as the number of regional R&D personnel, R&D expenditures, patent applications, patent authorizations, and invention patent authorizations.

We use model (3) to conduct an in-depth study of the mechanism of technological progress in reducing *PM*2.5 concentration through power market reform. From the results reported in Table 9, it can be seen that no matter what variable is used to characterize the progress and innovation of production technology, the implementation of power market reform can significantly promote technological progress and innovation at the level of 1%, thereby achieving a reduction in *PM*2.5 concentration. decline. We proved Hypothesis 3.

**Table 9:** The impact of power market reform on technological progress.

	R & D personnel	R & D spending	Patent applications	Patents granted	Invention patents granted
	(1)	(2)	(3)	(4)	(5)
*Year*_2015_*Power_*i*_	0.9113***	0.2128***	0.8699***	0.4143***	0.1378***
	(0.0417)	(0.0212)	(0.0417)	(0.0946)	(0.0210)
Control variables	YES	YES	YES	YES	YES
City fixed effect	YES	YES	YES	YES	YES
Year fixed effect	YES	YES	YES	YES	YES
Constant	0.8026**	0.9092***	0.2202	0.7430***	1.8623***
	(0.3083)	(0.2749)	(0.2312)	(0.2738)	(0.3470)
Adjusted R-squared	0.9435	0.9215	0.9327	0.9011	0.9289
Observations	851	851	851	851	851
Cities	92	92	92	92	92

### 5.3. Power consumption

Considering that the reform of China’s power market is mainly to reduce the industrial power price and increase the residential power price, and the increase of the residential power price will help curb the excessive consumption and waste of power in the residential sector, thus achieving the reduction of *PM*2.5 concentration, this paper uses model (3) to analyze the mechanism related to power consumption. In the column (1) of Table 10, this paper analyzes the mechanism of power consumption in the whole society in the process of power market reform affecting *PM*2.5 concentration, and finds that power market reform can indeed reduce power consumption, thereby achieving the reduction of *PM*2.5 concentration.

**Table 10:** Impact of power market reform on power consumption.

	Total	Per capita	Intensity
	(1)	(2)	(3)
*Year*_2015_*Power_*i*_	-0.3255***	-0.2378***	-0.6593***
	(0.0016)	(0.0077)	(0.0629)
Control variables	YES	YES	YES
City fixed effect	YES	YES	YES
Year fixed effect	YES	YES	YES
Constant	1.9664***	1.7455***	1.9023***
	(0.3965)	(0.2167)	(0.1662)
Adjusted R-squared	0.8994	0.8951	0.9077
Observations	851	851	851
Cities	92	92	92

Considering that the power consumption of the residential sector is related to the population, while the power consumption of the industrial sector is related to the economic output. In order to exclude the impact of population and economic output, we estimated the per capita power consumption and power consumption intensity in columns (2) and (3), respectively. In general, the reform of the electricity market can reduce the power consumption of the whole society, thereby reducing the emission of air pollutants. These findings are suggestive of support for the mechanism as mentioned in Hypothesis 4.

## 6. Heterogeneity analysis

Baseline regression studies the average effect of power market reform on *PM*2.5. On average, the implementation of power market reforms can reduce *PM*2.5 concentrations by 0.54%, thereby curbing air pollution. This part mainly analyzes the heterogeneity of *PM*2.5 concentration affected by power market reform from the perspective of region, *PM*2.5 concentration and population size, in order to further analyze the impact of power market reform on *PM*2.5 concentration.

### 6.1. Regional heterogeneity

The eastern region of China has developed economy, advanced production technology and relatively complete infrastructure construction, while the central and western regions are relatively backward in terms of economic development, production technology and infrastructure construction. In order to explore the impact of power market reform on *PM*2.5 concentration in different regions, we divide all samples into eastern, central and western regions, and compare and analyze the heterogeneity of the impact of power market reform on *PM*2.5 concentration in different regions through Table 11. It can be seen that the power market reform has the most significant inhibitory effect on *PM*2.5 concentration in the eastern region, while the inhibitory effect is weaker in the central and western regions. The possible reason is that the economy in the eastern region is relatively developed, the production technology is relatively advanced, the infrastructure construction is relatively complete, and the degree of marketization is relatively higher. The market-oriented reform of the power market can be efficiently implemented in the eastern region, which can effectively reduce the concentration of *PM*2.5.

**Table 11:** Regional heterogeneity.

	Eastern	Central	Western
	(1)	(2)	(3)
*Year*_2015_*Power_*i*_	-0.8090***	-0.4679	0.1657
	(0.2770)	(0.3007)	(0.2344)
Control variables	YES	YES	YES
City fixed effect	YES	YES	YES
Year fixed effect	YES	YES	YES
Constant	-2.0802	6.3774***	3.1101***
	(2.8873)	(1.5649)	(1.1078)
Adjusted R-squared	0.9218	0.9243	0.9622
Observations	252	287	312
Cities	33	29	30

### 6.2. Heterogeneity of air pollution levels

In order to study the heterogeneity of the impact of power market reform in areas with different air pollution levels, we divided the top 50% of the samples with higher *PM*2.5 concentrations as areas with severe air pollution, and the rest as areas with good air quality. From the results reported in Table 12, it can be found that power market reform has a more significant inhibitory effect in areas with higher *PM*2.5 concentrations. The possible reason is that, on the one hand, power market reform has a higher marginal effect in the process of directly affecting *PM*2.5 concentrations. On the other hand, in the process of power market reform indirectly affecting *PM*2.5 concentration, mechanisms such as efficiency improvement and technological innovation can also play a more effective role.

**Table 12:** Heterogeneity of air pollution levels.

	High	Low
	(1)	(2)
*Year*_2015_*Power_*i*_	-0.6333***	-0.4142
	(0.1744)	(0.7003)
Control variables	YES	YES
City fixed effect	YES	YES
Year fixed effect	YES	YES
Constant	-1.2844***	-1.1913
	(0.3360)	(0.7545)
Adjusted R-squared	0.9267	0.9112
Observations	431	420
Cities	47	45

### 6.3. Population size heterogeneity

Considering that the reform of the power market can reduce the excessive consumption of power in the residential sector to achieve the reduction of *PM*2.5 concentration, we grouped the cities according to the size of the population, and compared and analyzed the impact of the power market reform on *PM*2.5 in cities with different population sizes. From the results reported in Table 13, it can be seen that the power market reform has the most significant inhibitory effect on *PM*2.5 concentrations in cities with larger populations. This also confirms that the reform of the power market can ultimately reduce the concentration of *PM*2.5 by reducing excessive power consumption by residents.

**Table 13:** Population size heterogeneity.

	High	Low
	(1)	(2)
*Year*_2015_*Power_*i*_	-0.0560***	-0.0037
	(0.0185)	(0.0300)
Control variables	YES	YES
City fixed effect	YES	YES
Year fixed effect	YES	YES
Constant	-0.1138	-0.0789
	(0.1190)	(0.0822)
Adjusted R-squared	0.9176	0.9211
Observations	429	422
Cities	47	45

## 7. Conclusions and policy recommendations

China’s power industry is the industry that causes the most serious environmental pollution. Exploring the relationship between power market reform and *PM*2.5 concentration has become a topic of focus in the development of China’s power market and the implementation of policies such as "Beautiful China" and "Ecological Civilization Construction". This paper matches the precious industrial power price data of 100 cities in China from 2006 to 2018 with macroeconomic data, and conducts research based on the quasi-natural experiment of the marketization reform of the power industry in 2015. We use the DID model to analyze the impact and mechanism of the market-oriented reform of the power market on air pollution for the first time. The empirical research results show that the market-oriented reform of the power market can significantly reduce the severity of air pollution characterized by *PM*2.5 concentration, and this conclusion has also passed various robustness tests. The results of the mechanism analysis show that the market-oriented reform of the power market mainly reduces the level of air pollution by promoting the improvement of the operation efficiency of the power industry, technological progress and reduction of power consumption. Heterogeneity analysis shows that power market reform has a stronger inhibitory effect on *PM*2.5 in eastern regions, regions with severe air pollution, and regions with high population size. The research conclusions of this paper provide new perspectives and evidence for the governance and improvement of China’s air pollution, and also provide empirical support for the positive externalities of China’s power market reform.

Based on the research findings, this paper puts forward the following policy recommendations.

First, government departments should promulgate various policies to improve China’s power market reform while deepening the improvement of air pollution in the process of power market reform. Given that China’s power market reforms can curb air pollution. On the one hand, government departments should, through the promulgation of laws and regulations, strengthen the supervision of the reform process of China’s power market, and strengthen the penalties for violations in the process of power market reform. The government should also ensure the smooth and orderly reform of the power market, ensure that the market mechanism and price mechanism in the power market fully play their role, so as to play a positive externality role in improving the environment in the process of the power market reforming to marketization. On the other hand, in the process of promulgating policies and measures related to the reform of the power market, the government should also pay attention to complementing the air pollution prevention and control policies and reducing the conflicts in the process of policy implementation. The government should strive to realize the integration of power market reform policies and environmental pollution control policies, so as to maximize the restraint effect of power market reform on air pollution.

Second, government departments should also strengthen the promulgation of supporting measures in the process of implementing the power market reform policy, and promote the power market reform to play a more efficient role in the power market efficiency, technological progress and power consumption in the process of air pollution suppression. Due to the mechanism analysis, this paper finds that power market reform can restrain environmental pollution by improving the efficiency of power market operation, promoting technological progress and reducing power consumption. Therefore, government departments can encourage relevant industrial enterprises to increase investment in research and development or carry out technological innovation through tax relief, innovation subsidies and other supporting measures, so as to promote the reform of the power market to play a greater role in suppressing air pollution. On the other hand, government departments should set a more accurate residential power differential pricing model to suppress excessive power consumption and waste of power by residential departments. For example, improving the peak-valley power price and the step power price. Government departments can also improve residents’ awareness of energy conservation and environmental protection through publicity, education, and this will also help the power market reform to suppress air pollution.

Finally, each local government department should also formulate practical and feasible power market reform policies and air pollution control policies that are in line with their own development according to their own development conditions, so as to prevent the policy from being “one size fits all”. Specifically, on the one hand, because different regions and cities have completely different levels of economic development, industrialization, infrastructure construction, population size, and air pollution severity, each local government should formulate power market reform policies and measures and air pollution control goals that are in line with their own development reality and are conducive to their own long-term development. If local governments ignore their own development reality and copy the policy measures and goals of other regions, the implementation of policies may run counter to the policy goals. On the other hand, given that power market reform has a better effect on curbing air pollution in eastern regions, regions with severe air pollution, and regions with larger populations, the central government should not only further support the reform of the power market in these regions, so as to promote the reform of the power market to suppress air pollution more efficiently. At the same time, the central government should also pay attention to the reform of the power market in the central and western regions, promote the smooth completion of the power market reform in the central and western regions through funds and policies, and play a greater role in curbing air pollution.

With the introduction of air pollution prevention and control policy goals such as "Beautiful China" and "Ecological Civilization Construction", China’s air pollution control is also in full swing. As the industry with the largest energy consumption and the largest emission of air pollutants in China, the market-oriented reform of the power market can improve the operation efficiency and greatly reduce the emission of air pollution from the source, which is the key means to improve the air quality in China. However, the existing literature has insufficient understanding of the impact and mechanism of power market reform on air pollution, and lacks rigorous empirical analysis. By studying the impact and mechanism of power market reform on air pollution, this paper not only finds a new perspective and policy reference for China’s air pollution control, but also provides a realistic basis for the positive externality of power market reform. It should be noted that due to the difficulty in obtaining *PM*2.5 concentration data related to the power industry, this paper only roughly estimates the causal relationship between power market reform and air pollution. The follow-up research plan is mainly to collect and obtain more sufficient *PM*2.5 concentration data in China’s power industry, in order to more rigorously and accurately evaluate the impact of power market reform on air pollution.

## Supporting information

S1 Data (DTA)
